# Effects of betaine on growth performance and colonic microbiota in weaned piglets

**DOI:** 10.3389/fvets.2026.1937273

**Published:** 2026-10-28

**Authors:** Haichao Wang, Zhengge Zhao, Tong Pang, Dongmei Lin, Jianmin Chai

**Affiliations:** 1 School of Life Sciences and Food Engineering, Hebei University of Engineering, Handan, China; 2 School of Animal Science and Technology, Foshan University, Foshan, China

**Keywords:** weaned piglets, betaine, growth performance, gut microbiota, 16S rRNA gene sequencing

## Abstract

This study investigated the effects of dietary betaine supplementation on growth performance, diarrhea incidence, and the colonic microbiota of weaned piglets. A total of newly weaned 150 Duroc × Landrace × Yorkshire piglets were randomly assigned to three dietary treatments: a control diet or diets supplemented with 1,250 or 2,500 mg/kg betaine. Growth performance and diarrhea incidence were evaluated, and colonic microbial composition was analyzed by 16S rRNA gene sequencing. The functional potential of the microbiota was further predicted using PICRUSt2 and Tax4Fun2. The results showed that, compared with the control group, both betaine-supplemented groups significantly increased average daily gain (ADG) and reduced feed conversion ratio (FCR) and diarrhea incidence (*p* < 0.05). Exploratory random forest analysis identified several candidate genera contributing to group discrimination, including *Prevotellaceae_NK3B31_group*, *Oscillibacter*, *Oscillospira*, and *Ruminococcus_torques_group*. Specifically, low-dose betaine supplementation altered the relative abundances of several genera, including *Prevotellaceae_NK3B31_group*, *Oscillibacter*, *Oscillospira*, and *Ruminococcus_torques_group*. Functional prediction further suggested that low-dose betaine was associated with enrichment of predicted KEGG pathways related to energy metabolism, including the citrate cycle (TCA cycle) (ko00020), butanoate metabolism (ko00650), and folate biosynthesis (ko00790). Overall, dietary betaine supplementation improved growth performance and reduced diarrhea incidence in weaned piglets. These phenotypic improvements were accompanied by changes in selected colonic bacterial taxa and predicted microbial functions. However, because microbial metabolites, SCFAs, intestinal barrier function, and immune indicators were not directly measured, the microbiota-related findings should be interpreted as associative and require further validation.

## 1 Introduction

Weaning stress is one of the most critical challenges affecting the health and productivity of piglets in modern swine production. Abrupt maternal separation, dietary transition from milk to solid feed, environmental changes, and social regrouping collectively induce physiological and psychological stress, leading to reduced feed intake, impaired intestinal morphology, increased intestinal permeability, immune dysfunction, oxidative stress, and a high incidence of post-weaning diarrhea (1–3). Moreover, weaning stress profoundly disrupts the gut microbial ecosystem, resulting in microbial dysbiosis, reduced colonization resistance, and the expansion of opportunistic pathogens, which further aggravate intestinal inflammation and metabolic disorders (1, 4). Since the gut microbiota plays indispensable roles in nutrient metabolism, short-chain fatty acid production, maintenance of intestinal barrier integrity, and immune homeostasis, nutritional strategies that alleviate weaning stress by modulating the gut microbiota have attracted increasing attention as effective alternatives to reduce post-weaning diarrhea and antibiotic use in pig production (5, 6).

Betaine is a naturally occurring quaternary ammonium compound that functions as both a methyl donor and an osmoprotectant (7). Previous studies have demonstrated that betaine participates in protein and lipid metabolism, enhances antioxidant capacity, alleviates stress responses, and maintains cellular osmotic balance (8). In swine nutrition, dietary betaine supplementation has been shown to improve nutrient digestibility, enhance growth performance, and reduce post-weaning diarrhea (9, 10). Moreover, betaine may regulate intestinal health by modulating gut microbial composition and fermentation processes, promoting beneficial bacteria, strengthening intestinal barrier function, and regulating host immune responses (8, 11). However, current studies on betaine mainly focus on growth performance and nutrient metabolism, while its effects on colonic microbial structure, functional characteristics, and microbe–function interactions in weaned piglets remain poorly understood.

Therefore, the present study aimed to evaluate the effects of dietary betaine supplementation (1,250 and 2,500 mg/kg) on growth performance, diarrhea incidence, and colonic microbial profiles in weaned piglets. We further used PICRUSt2 and Tax4Fun2 to explore whether changes in predicted microbial functions were associated with betaine supplementation. We hypothesized that dietary betaine supplementation would improve growth performance and reduce diarrhea incidence, and that these phenotypic responses might be accompanied by alterations in colonic microbial composition and predicted functional potential. Rather than establishing a causal microbiota-mediated mechanism, this study was designed to identify microbial and predicted functional features potentially associated with the phenotypic effects of betaine supplementation.

## 2 Materials and methods

### 2.1 Animals and experimental design

The experimental protocol was approved by the Animal Care and Use Committee of Hebei University of Engineering (Handan, China). The animal experiments were conducted in strict adherence to the guidelines outlined in the Care and Use of Animals (BER-YXY-2024016).

A total of 150 healthy 28-day-old Duroc × Landrace × Yorkshire weaned piglets with similar body weights and genetic background were selected and assigned to three dietary treatment groups using a completely randomized design: a control group, a low-dose group, and a high-dose group. Each treatment consisted of five replicates with 10 piglets per replicate. Piglets in the control group were fed a basal diet, while those in the low- and high-dose groups received the basal diet supplemented with 1,250 mg/kg and 2,500 mg/kg betaine, respectively. These supplementation levels were selected based on previous studies in weaned piglets and growing pigs showing that dietary betaine at approximately 1,250–2,500 mg/kg can improve growth performance, nutrient utilization, antioxidant status, and intestinal health-related outcomes. In addition, these levels are within practical supplementation ranges used in pig nutrition studies and represent low and high supplementation levels for evaluating dose-related responses (7, 9, 10). All diets were formulated based on corn–soybean meal and met the nutrient requirements of weaned piglets according to NRC (2012) (12). The ingredient composition and nutrient levels of the basal diet were presented in Supplementary Table 1. The experiment lasted 35 days, including a 5-day adaptation period and a 30-day experimental period. Piglets were fed three times a day (06:00, 12:00, and 18:00) with ad libitum access to feed and water. The ambient temperature was maintained at 25–30 °C under standard ventilation conditions.

### 2.2 Growth performance measurements

Body weight was recorded at the beginning and end of the experiment in the morning before feeding. Daily feed consumption was recorded for each pen to calculate average daily gain (ADG), average daily feed intake (ADFI), and feed conversion ratio (FCR). Faecal score was evaluated every day during the experimental period on a scale from 1 to 4 according to the degree of consistency (1 = firm and shaped; 2 = soft and shaped; 3 = loose; 4 = watery) developed by Pedersen and Toft with faecal scores 1 and 2 classified as “normal” faeces and scores 3 and 4 as “diarrhea” (13). Diarrhea incidence was calculated as follows: diarrhea incidence (%) = 100 × *n*/(*N* × day), where n is the sum of daily diarrhea cases across the experimental period in a pen, *N* is the total number of piglets in the pen, and day is the number of experimental days (10).

### 2.3 Sample collection

At 12 h before the end of the feeding trial, all piglets were fasted overnight. On the following morning, after recording final body weights, one representative piglet per pen, with body weight closest to the pen average, was selected for microbiome analysis and sample collection. This resulted in five biological replicates per treatment and fifteen piglets in total. All selected piglets were sedated with electrical stunning followed by euthanasia with an overdose of pentobarbital intravenously via the ear vein as described by Moeser et al. (14). After loss of consciousness was confirmed, all piglets were sacrificed by exsanguination. After dissection, the entire colon was immediately separated, and colonic chyme samples were aseptically collected into sterile cryotubes, snap-frozen in liquid nitrogen, and stored at −80 °C until further analysis for gut microbial high-throughput sequencing.

### 2.4 DNA extraction and 16S rRNA gene sequencing

Total genomic DNA was extracted from colonic chyme samples using the E. Z. N. A.™ Mag-Bind Soil DNA Kit (Omega Bio-Tek, Norcross, GA, United States) following the manufacturer’s instructions. The V3–V4 hypervariable region of the bacterial 16S rRNA gene was amplified using the universal primers CCTACGGGNGGCWG CAG (forward) and GACTACHVGGGTATCTAATCC (reverse). PCR was performed in a 30-μL reaction mixture containing 2 μL of microbial DNA (10 ng/μL), 1 μL of each primer (10 μM), 15 μL of 2 × Hieff® Robust PCR Master Mix (Yeasen, China), and ddH₂O to a final volume of 30 μL. PCR was performed on an Applied Biosystems 9700 thermal cycler (United States) using the following program: initial denaturation at 95 °C for 3 min; 5 cycles of 95 °C for 30 s, 45 °C for 30 s, and 72 °C for 30 s; 20 cycles of 95 °C for 30 s, 55 °C for 30 s, and 72 °C for 30 s; and a final extension at 72 °C for 5 min. PCR products were verified by 2% (w/v) agarose gel electrophoresis in TBE buffer stained with ethidium bromide and visualised under UV light, and purified using Hieff NGS™ DNA Selection Beads (Yeasen, China). Libraries were constructed by Sangon Biotech (Shanghai, China) using universal Illumina adaptors and indices, quantified with a Qubit 4.0 fluorometer, and quality-controlled using an Agilent 2100 bioanalyzer. Sequencing was performed on the Illumina MiSeq platform with paired-end 300-bp reads.

Since the raw sequencing data were paired-end reads containing barcode sequences as well as primer and adapter sequences, cutadapt (version 1.18) (15) was firstly used to remove the sequencing primer adapter from the 3′ end of Read1 (AGATCGGAAGAGCACACGTCTG AACTCCAGTCA) and from the 3′ end of Read2 (AGATCGGAAGA GCGTCGTGTAGGGAAAGAGTGT). Paired-end reads were merged based on overlap using PEAR (version 0.9.8) (16), and then demultiplexed according to barcode sequences. Quality control was performed using PRINSEQ (version 0.20.4) (17): a 10 bp sliding window was applied to trim bases with an average quality score below 20 from the 3′ end, and sequences containing ambiguous bases (N), as well as short and low-complexity sequences, were filtered out. High-quality sequences were clustered into operational taxonomic units (OTUs) at 97% similarity using USEARCH (version 11.0.667) (18), and chimeric sequences were removed during clustering. Taxonomic assignment of representative OTU sequences was performed using the RDP classifier (version 2.12) against the RDP 16S rRNA database (19). After quality filtering, the number of high-quality sequences per sample ranged from 47,479 to 69,748 reads, with an average of 56,213 reads per sample. Before performing alpha diversity and beta diversity analysis, we adopted a rarefaction strategy to standardize all samples to a uniform sequencing depth of 47,000 reads per sample — this value was set according to the minimum sequence count across all samples, ensuring that no sample was excluded during the standardization process. Functional predictions were conducted using PICRUSt2 (version 2.5.2) (20) and Tax4Fun2 (version 1.1.5) (21).

### 2.5 Statistical analysis

Growth performance parameters, including average daily gain (ADG), average daily feed intake (ADFI), and feed conversion ratio (FCR), were calculated at the pen level, and the pen was considered the experimental unit. Pen-level diarrhea incidence was analyzed together with growth performance parameters using one-way analysis of variance (ANOVA). Before ANOVA, the assumptions of normality and homogeneity of variance were checked. The Shapiro–Wilk test showed no significant deviation from normality in each group (*p* > 0.05), and Levene’s test indicated homogeneity of variance (*p* = 0.295). In addition, arcsine square-root transformation of the percentage data produced consistent results (10). Individual piglet represented as the experiment unit for the 16S sequencing data. Alpha diversity indices, including the Richness index (also referred to as the observed-species index, S.obs), Chao1 index (S.chao1), Shannon index, and Simpson index, were compared among groups using the Kruskal–Wallis test. Pairwise comparisons were performed using the Wilcoxon rank-sum test. Beta diversity was assessed based on the Bray-Curtis distance matrix. Principal coordinate analysis (PCoA) was performed to visualize the similarities or differences in microbial community composition among samples, and permutational multivariate analysis of variance (PERMANOVA, also known as Adonis) was used to test the statistical significance of group-wise differences. Differential microbial taxa among groups were identified using the Kruskal-Wallis test, followed by pairwise Wilcoxon rank-sum tests where appropriate, and the results are presented as mean ± standard error (SE). For multiple comparisons of bacterial taxa and predicted KEGG pathways, *p* values were adjusted using the Benjamini-Hochberg false discovery rate (FDR) method. FDR-adjusted *p* values < 0.05 were considered statistically significant. Additionally, random forest analysis was performed at the genus level as an exploratory approach to identify genera contributing to group discrimination among treatments. The importance of each genus was evaluated using mean decrease accuracy (MDA). The top 20 genera were displayed to visualize the highest-ranked variables based on importance scores, rather than as a strict biological threshold. Given the limited number of microbiome samples, the random forest results were interpreted as exploratory and require further validation in larger independent cohorts. Spearman correlation analysis was conducted to explore co-variation patterns between differential microbial genera and predicted KEGG pathways. Because both bacterial genera and predicted KEGG pathways were derived from the same 16S rRNA gene sequencing dataset, this analysis was considered exploratory rather than confirmatory. Correlations with |r| > 0.5 and FDR-adjusted *p* < 0.05 were considered significant. The networks were visualized using Cytoscape software (version 3.10.3). Figures were prepared using Adobe Illustrator 2025 for layout and visual optimization.

## 3 Results

### 3.1 Effects of betaine on growth performance and diarrhea rate of weaned piglets

Dietary betaine supplementation significantly affected the growth performance of weaned piglets (Figure 1). Compared with the Control group, both the Low_Dose and High_Dose betaine groups exhibited significantly increased average daily gain (ADG) (*p* < 0.05), as well as significantly decreased feed conversion ratio (FCR) and diarrhea rate (*p* < 0.05). Moreover, the diarrhea incidence in the High_Dose group was significantly lower than that in the Low_Dose group (*p* < 0.05). There was no significant difference in average daily feed intake (ADFI) among all groups (*p* > 0.05).

**Figure 1:**
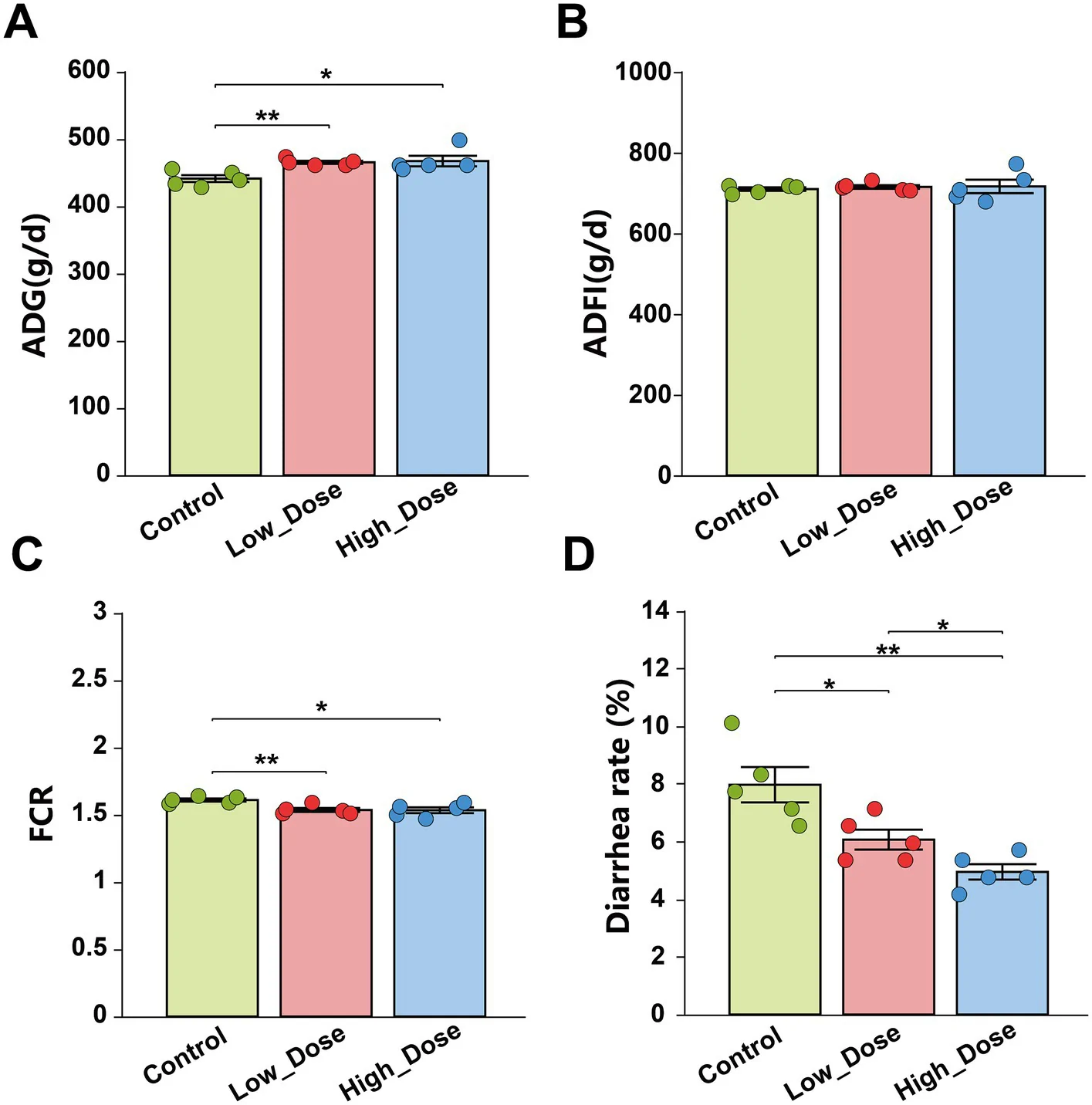
Effects of dietary betaine supplementation on growth performance and diarrhea rate of weaned piglets. **(A)** Average daily gain (ADG); **(B)** average daily feed intake (ADFI); **(C)** feed conversion ratio (FCR); **(D)** diarrhea incidence. Values are presented as mean ± standard error of the mean (SEM), with each treatment group comprising five replicate pens (*n* = 5 per group). Control, basal diet; Low_Dose, basal diet supplemented with 1,250 mg/kg betaine; High_Dose, basal diet supplemented with 2,500 mg/kg betaine. Asterisks indicate significant differences among groups based on one-way ANOVA (*p < 0.05, **p < 0.01).

### 3.2 Effects of betaine on colonic microbial diversity and composition in weaned piglets

To assess the effect of dietary betaine supplementation on the overall architecture of the intestinal microbiota, the alpha and beta diversity of colonic microbial communities were examined (Supplementary Figure 1). Regarding alpha diversity, no significant differences were observed among the three treatment groups in terms of Observed species (S.obs), Chao1 richness estimator, Shannon index, or Simpson index (*p* > 0.05). This indicated that dietary betaine supplementation did not significantly affect the alpha diversity of the colonic microbiota, including both species richness and community evenness. Likewise, principal coordinate analysis (PCoA) based on Bray–Curtis dissimilarity did not reveal a distinct separation of microbial community structures across groups (Supplementary Figure 1B). PERMANOVA (Adonis) confirmed that beta diversity did not differ significantly among the three groups (*p* > 0.05), suggesting that betaine supplementation failed to induce substantial alterations in the overall colonic microbial community structure.

To further characterize the taxonomic composition of the colonic microbiota, the relative abundances of dominant bacterial taxa at the phylum and genus levels were analyzed (Figure 2). At the phylum level, Firmicutes was the predominant phylum in all groups, followed by Proteobacteria, Bacteroidota, and Actinobacteriota, whereas other phyla, including Desulfobacterota, Spirochaetota, Cyanobacteria, Patescibacteria, and Campylobacterota, were present at relatively low abundances. At the genus level, the dominant genera included *Lactobacillus*, *Escherichia-Shigella*, *Clostridium_sensu_stricto_1*, *Agathobacter*, *Faecalibacterium*, *Lachnospira*, *norank_Clostridia_UCG-014*, *Dorea*, *Subdoligranulum*, and *Olsenella*. These genera constituted the major members of the colonic microbiota, and the overall taxonomic composition was broadly comparable among the three treatment groups.

**Figure 2:**
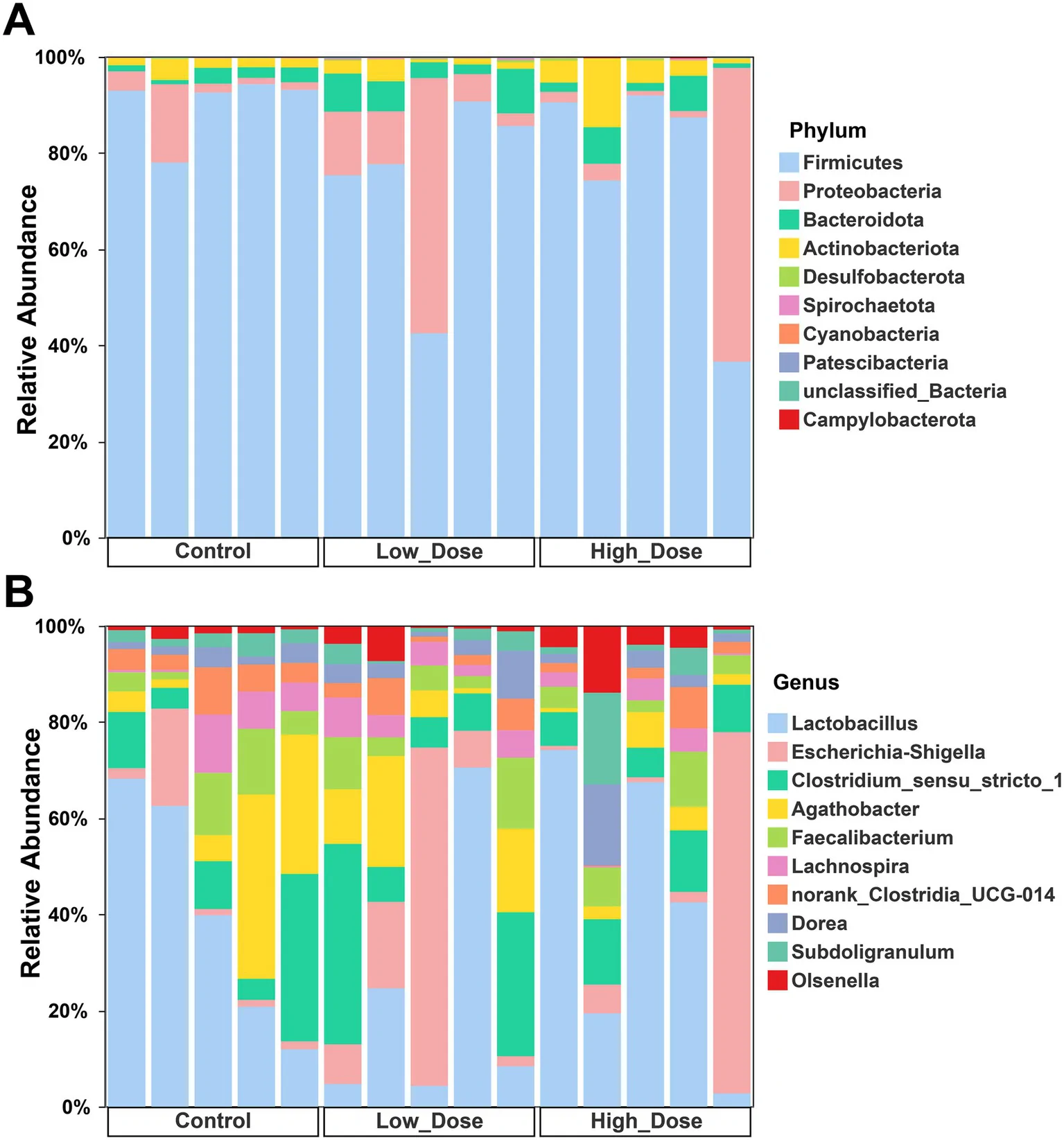
Colonic microbial community composition in piglets fed with different levels of betaine supplementation. **(A)** Phylum-level stacked barplots showing the composition and relative abundance distribution of the dominant bacterial phyla (top 10 in relative abundance) across different treatment groups (Control, Low_Dose, and High_Dose). The x-axis represents the treatment groups, and the y-axis represents relative abundance (%). Different colors indicate different phyla. **(B)** Genus-level stacked barplots showing the composition and relative abundance distribution of the dominant bacterial genera (top 10 in relative abundance) across different treatment groups. Different colors indicate different genera.

### 3.3 Candidate discriminatory genera identified by exploratory random forest analysis

As an exploratory approach, a random forest model was applied to identify genera contributing to group discrimination among treatments. Based on the mean decrease accuracy (MDA), a total of 20 genera were identified as important contributors to group discrimination (Figure 3A). Among these taxa, *Prevotellaceae_NK3B31_group*, *Oscillospira*, *Oscillibacter*, *Ruminococcus_torques_group*, and *norank_UCG-010* showed relatively high importance scores, suggesting that these genera contributed to treatment discrimination in the exploratory model. The relative abundances of the top 20 candidate discriminatory genera were further compared among groups, and differences with FDR-adjusted *p* < 0.05 were considered significant. The results showed that 12 genera (Figure 3B–M) exhibited significant differences among the different treatment groups (FDR-adjusted *p* < 0.05). Compared with the Control group, the Low_Dose group exhibited significantly increased relative abundances of *Prevotellaceae_NK3B31_group*, *norank_Peptococcaceae*, *unclassified_Bacteroidales*, *Oscillospira*, *Oscillibacter*, *norank_UCG-010*, and *unclassified_Selenomonadaceae*, whereas *Ruminococcus_torques_group* was significantly decreased in the Low_Dose group (FDR-adjusted *p* < 0.05). Compared to the Low_Dose, *Prevotellaceae_NK3B31_group*, *norank_Peptococcaceae*, *unclassified_Bacteroidales*, *Fibrobacter*, and *Eubacterium_xylanophilum_group* were significantly decreased in the High_Dose group (FDR-adjusted *p* < 0.05), while *Syntrophococcus* was significantly increased in the High_Dose group (FDR-adjusted *p* < 0.05). In addition, compared with the Control group, *norank_Bradymonadales* was significantly increased in the High_Dose group (FDR-adjusted *p* < 0.05), suggesting that this taxon may be more sensitive to high-dose betaine supplementation. In contrast, several genera identified by the random forest model, including *Anaerovibrio*, *Mogibacterium*, *Solobacterium*, *Peptococcus*, *Alistipes*, and *unclassified_Firmicutes*, showed no significant differences among groups (Supplementary Figure 2).

**Figure 3:**
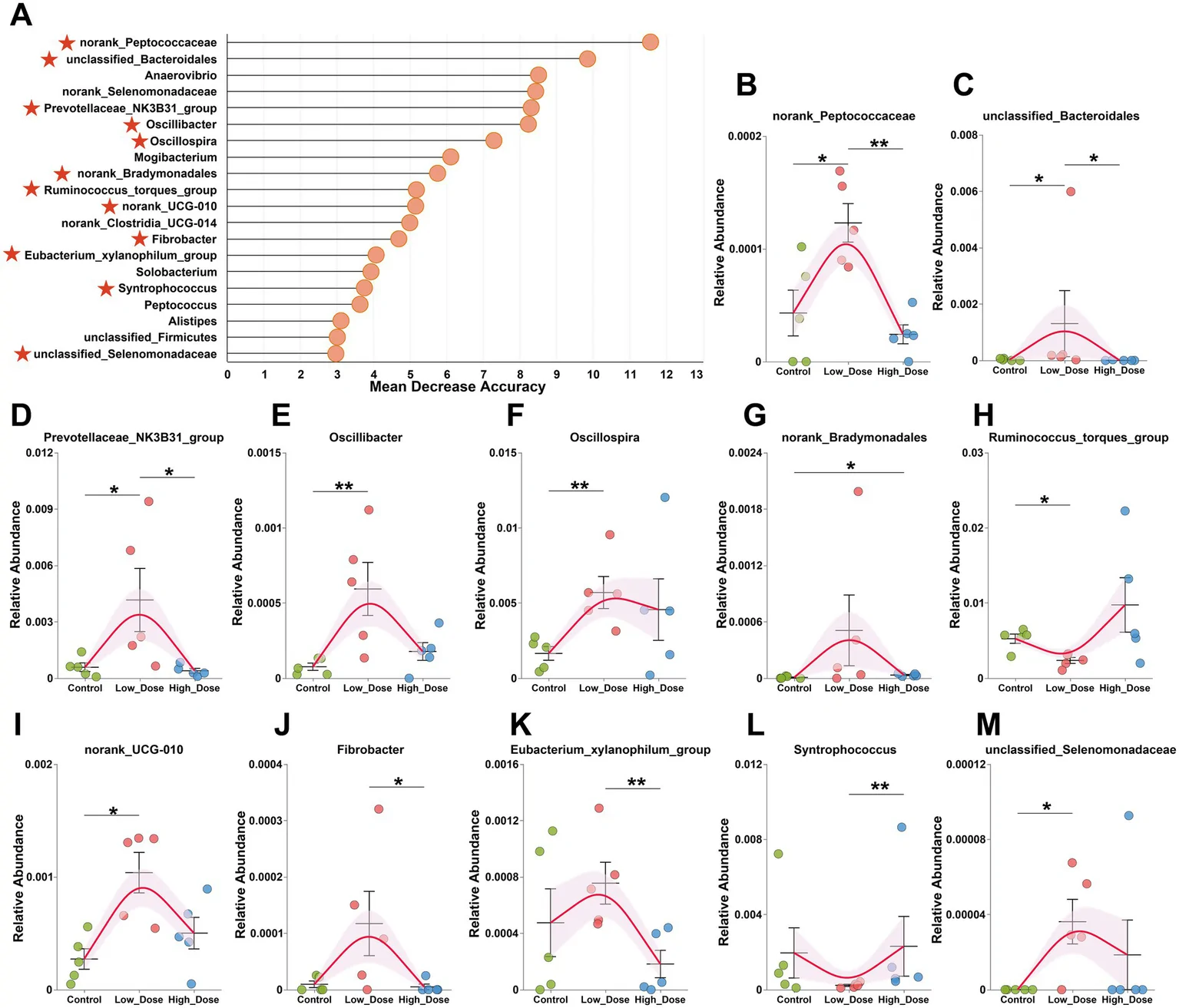
Candidate discriminatory genera identified by exploratory random forest analysis and their relative abundances across groups. **(A)** Mean decrease accuracy (MDA) values of the top 20 genera ranked by the exploratory random forest model, indicating their contribution to group discrimination among Control, Low_Dose, and High_Dose groups. **(B–M)** Boxplots showing the relative abundances of representative candidate discriminatory genera selected from the random forest analysis. Data are presented as mean ± standard error (SE). Statistical significance among groups was determined using pairwise Wilcoxon rank-sum tests with Benjamini-Hochberg FDR correction. Asterisks indicate significant differences (*FDR-adjusted *p* < 0.05, **FDR-adjusted *p* < 0.01). The top 20 genera were displayed to visualize the highest-ranked variables based on MDA rather than as a strict biological threshold.

### 3.4 Correlation of colonic microbial genera in weaned piglets

Spearman correlation analysis revealed interaction patterns among differential bacterial genera in the colonic microbiota (Figure 4). Several differential bacterial genera exhibited significant positive correlations with each other. Specifically, *Oscillibacter* showed significant positive correlations with *Oscillospira* and *norank_UCG-010*, while *Oscillospira* was also positively correlated with *unclassified_Selenomonadaceae* and *Eubacterium_xylanophilum_group* (FDR-adjusted *p* < 0.05). In addition, *Prevotellaceae_NK3B31_group* was positively correlated with *Oscillibacter*, *Oscillospira*, *norank_UCG-010*, and *norank_Bradymonadales*, but negatively correlated with *Syntrophococcus* and *Ruminococcus_torques_group* (FDR-adjusted *p* < 0.05). In contrast, *Ruminococcus_torques_group* was positively correlated with *Peptococcus* and *Solobacterium*, but negatively correlated with *Prevotellaceae_NK3B31_group*, *Oscillibacter*, and *Oscillospira* (FDR-adjusted *p* < 0.05).

**Figure 4:**
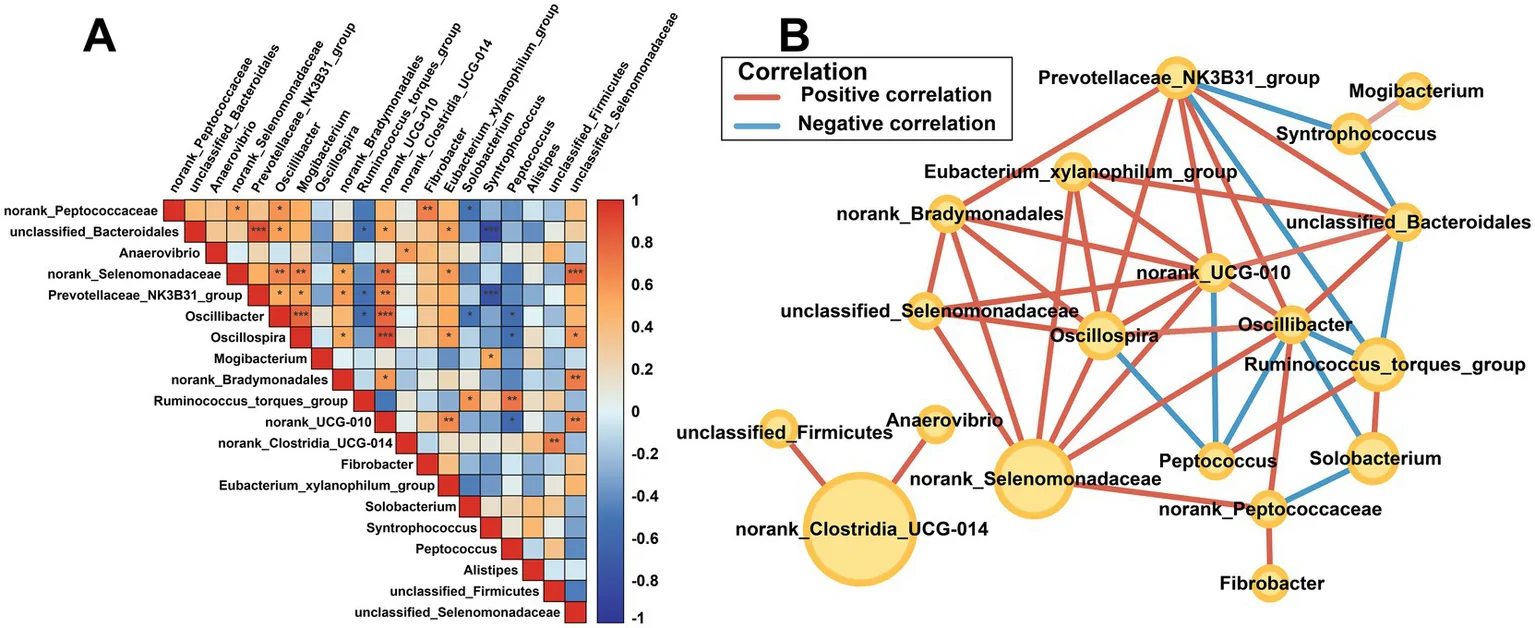
Association patterns among differential colonic bacterial genera in weaned piglets. **(A)** Heatmap showing Spearman correlations among the top 20 candidate discriminatory genera selected from the exploratory random forest analysis. The color gradient from blue to red represents Spearman’s r values from negative to positive correlations, with red indicating positive correlations and blue indicating negative correlations. Asterisks within each cell denote significant correlations between genera (*FDR-adjusted *p* < 0.05, **FDR-adjusted *p* < 0.01, ***FDR-adjusted *p* < 0.001). **(B)** Co-occurrence network of bacterial genera constructed based on Spearman correlation analysis (|r| > 0.5, FDR-adjusted *p* < 0.05). Each node represents a bacterial genus, with red and blue edges indicating positive and negative associations, respectively. Node size is proportional to the mean relative abundance of the corresponding genus across all samples.

### 3.5 Predicted functional profiles of colonic microbiota under betaine supplementation

To further investigate the functional potential of the gut microbiota under dietary betaine supplementation, PICRUSt2 and Tax4Fun2 were used to predict KEGG Level 3 functional profiles based on 16S rRNA gene sequencing data (Figure 5). The PICRUSt2 results showed that, compared with the Control group, the Low_Dose group exhibited significantly higher relative abundances in several KEGG Level 3 functional pathways, including folate biosynthesis (ko00790), lipoic acid metabolism (ko00785), lipopolysaccharide biosynthesis (ko00540), and citrate cycle (ko00020) (FDR-adjusted *p* < 0.05). In the Low_Dose vs. High_Dose comparison, the High_Dose group showed significantly higher abundances of nicotinate and nicotinamide metabolism (ko00760) and fructose and mannose metabolism (ko00051) (FDR-adjusted *p* < 0.05). Tax4Fun2 prediction results further indicated that the Control group exhibited significantly lower abundances of butanoate metabolism (ko00650), arachidonic acid metabolism (ko00590), and pentose and glucuronate interconversions (ko00040) compared with the Low_Dose group (FDR-adjusted *p* < 0.05). Overall, dietary betaine supplementation was associated with changes in selected predicted KEGG Level 3 functional profiles of the gut microbiota.

**Figure 5:**
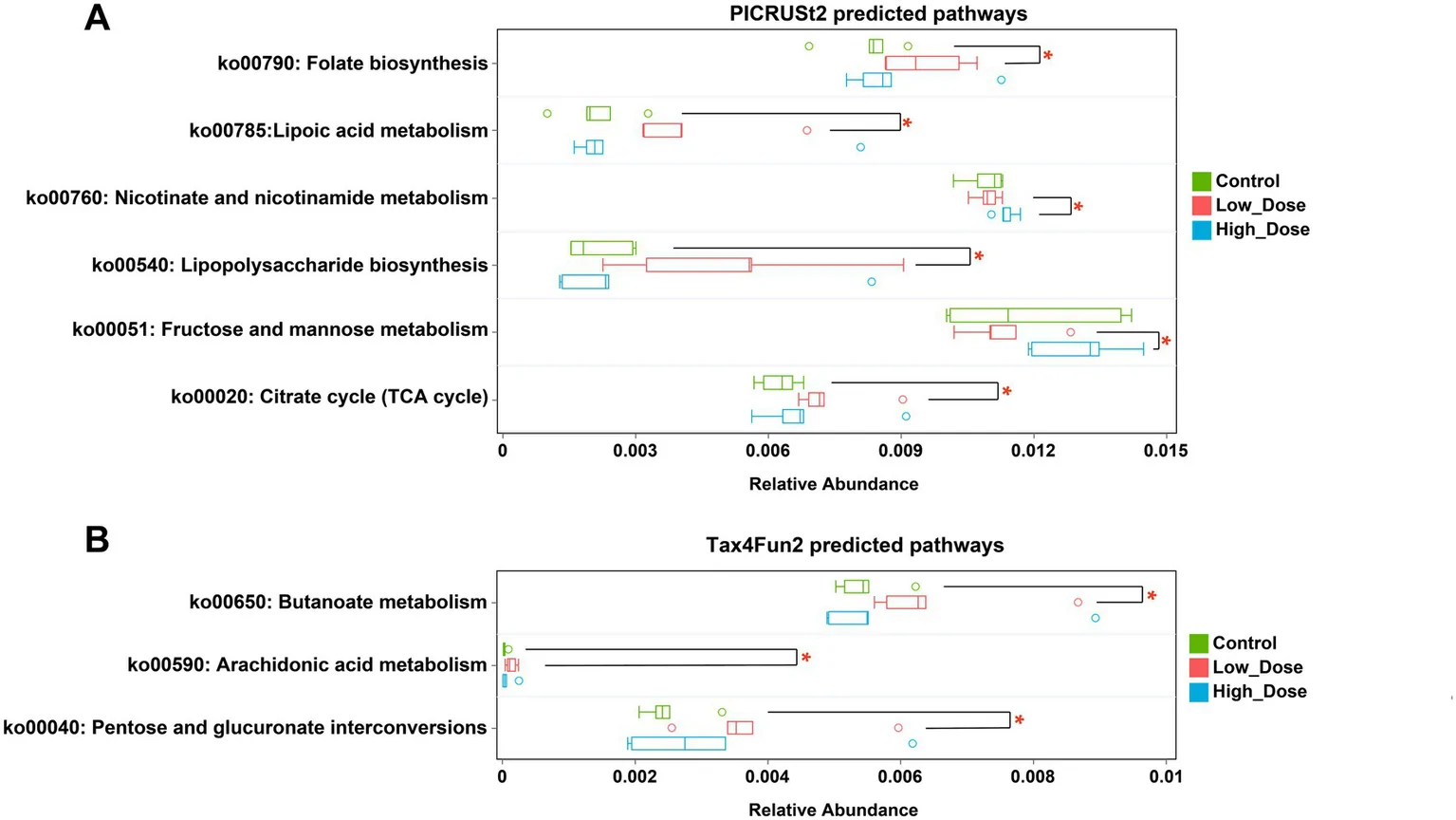
Predicted KEGG Level 3 functional profiles inferred by PICRUSt2 and Tax4Fun2. **(A)** Differential predicted KEGG Level 3 pathways inferred by PICRUSt2 based on 16S rRNA gene sequencing data. **(B)** Differential predicted KEGG Level 3 pathways inferred by Tax4Fun2 based on 16S rRNA gene sequencing data. Relative abundances of selected predicted KEGG Level 3 pathways are shown as boxplots. Data are presented as mean ± standard error (SE). Statistical differences among groups were evaluated using Wilcoxon rank-sum tests with Benjamini-Hochberg FDR correction. Significance is indicated as *FDR-adjusted *p* < 0.05.

### 3.6 Exploratory network analysis of co-variation between colonic microbiota and predicted KEGG functions

Spearman correlation analysis was performed to explore co-variation patterns between differential colonic bacterial genera and predicted KEGG pathways. Because the predicted functional profiles were inferred from the same 16S rRNA gene sequencing dataset used for taxonomic profiling, this analysis was interpreted as exploratory rather than as direct mechanistic evidence. The results showed that several genera co-varied with selected predicted KEGG pathways (Figure 6). Among them, *norank_Selenomonadaceae*, *norank_UCG-010*, and *unclassified_Selenomonadaceae* were all positively associated with predicted butanoate metabolism (ko00650), pentose and glucuronate interconversions (ko00040), citrate cycle (TCA cycle) (ko00020), and lipopolysaccharide biosynthesis (ko00540) (FDR-adjusted *p* < 0.05). Furthermore, *norank_UCG-010* and *unclassified_Selenomonadaceae* were additionally positively associated with predicted arachidonic acid metabolism (ko00590) and lipoic acid metabolism (ko00785), while *unclassified_Selenomonadaceae* also showed a positive correlation with folate biosynthesis (ko00790) (FDR-adjusted *p* < 0.05). In addition to the above core cluster, *norank_Peptococcaceae* was significantly positively associated with ko00540 and ko00020; both *Oscillibacter* and *Oscillospira* were positively associated with ko00020, and *Oscillospira* was additionally positively associated with ko00540 (FDR-adjusted *p* < 0.05). Moreover, *norank_Bradymonadales* was positively associated with ko00790 and ko00040, while *Syntrophococcus* was specifically significantly positively associated with predicted nicotinate and nicotinamide metabolism (ko00760) (FDR-adjusted *p* < 0.05).

**Figure 6:**
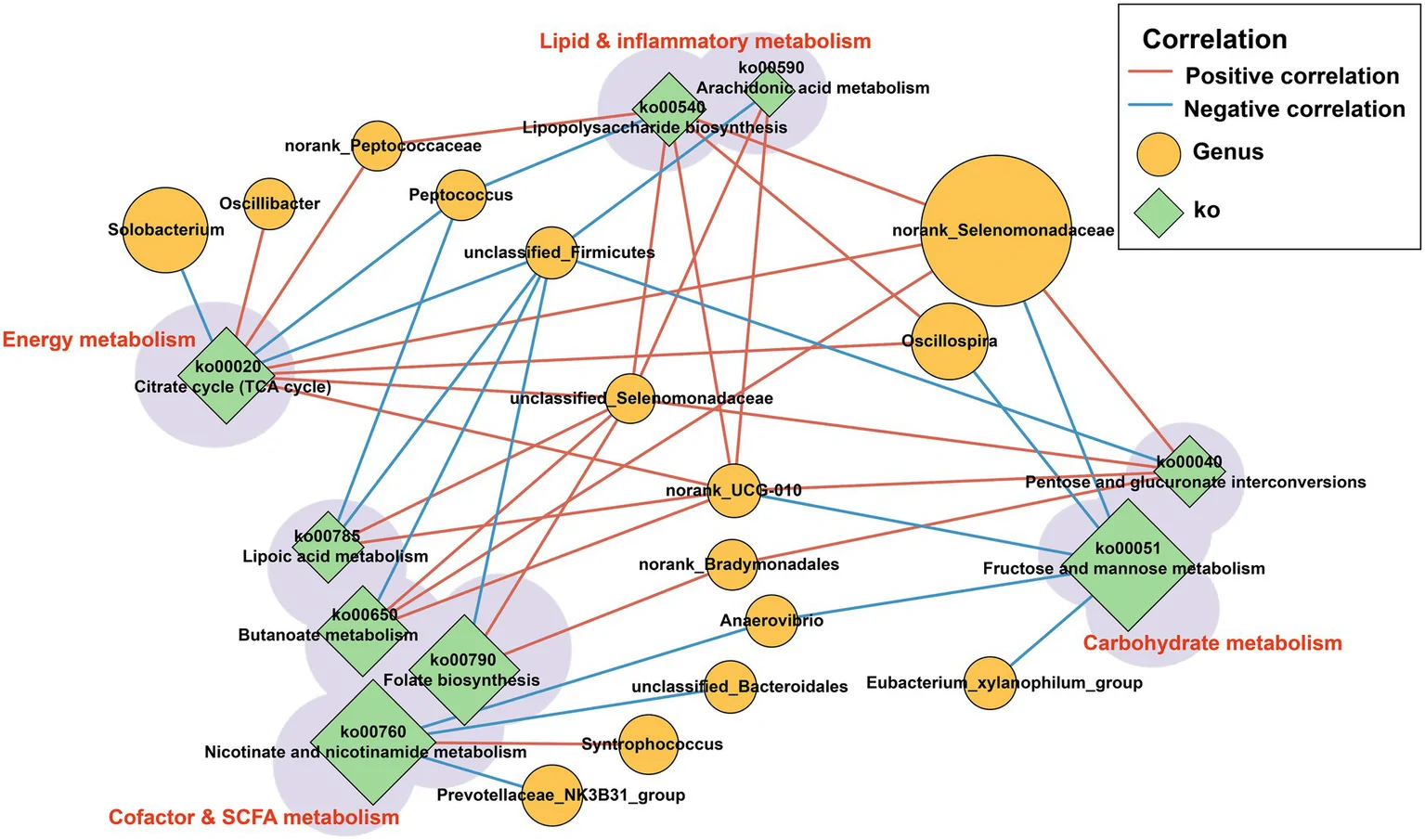
Exploratory correlation network showing co-variation patterns between bacterial genera and predicted KEGG Level 3 pathways in the colon of weaned piglets. Nodes represent bacterial genera and predicted KEGG Level 3 pathways. The size of each node is proportional to its mean relative abundance across all samples. Edges indicate associations between microbial taxa and predicted functional pathways (|*r*| > 0.5, FDR-adjusted *p* < 0.05). Red and blue lines represent positive and negative associations, respectively. Because bacterial genera and predicted KEGG pathways were derived from the same 16S rRNA gene sequencing dataset, this network should be interpreted as exploratory rather than as independent mechanistic evidence.

Conversely, certain genera showed negative associations with selected predicted pathways. Regarding fructose and mannose metabolism (ko00051), *Anaerovibrio*, *Eubacterium_xylanophilum_group*, *norank_Selenomonadaceae*, *norank_UCG-010*, and *Oscillospira* all showed significant negative correlations (FDR-adjusted *p* < 0.05). For ko00760, *Anaerovibrio*, *Prevotellaceae_NK3B31_group*, and *unclassified_Bacteroidales* were negatively correlated, whereas *Syntrophococcus* was positively correlated with this pathway, suggesting different co-variation patterns among genera within the same predicted functional module (FDR-adjusted *p* < 0.05). Additionally, *Peptococcus* and *Solobacterium* were negatively correlated with ko00020, and *Peptococcus* also showed negative correlations with ko00785 and ko00540 (FDR-adjusted *p* < 0.05). Notably, *unclassified_Firmicutes* exhibited the broadest negative correlation pattern, being significantly inversely associated with ko00590, ko00790, ko00040, ko00020, ko00785, and ko00650 (FDR-adjusted *p* < 0.05).

## 4 Discussion

The present study showed that dietary supplementation with 1,250 and 2,500 mg/kg betaine significantly increased the average daily gain (ADG), improved feed conversion ratio (FCR), and reduced diarrhea incidence in weaned piglets. These findings were consistent with those reported by Wang et al. (10), who demonstrated that increasing dietary betaine levels (0, 1,250, and 2,500 mg/kg) linearly enhanced ADG while improving FCR and reducing the incidence of diarrhea (10). However, betaine supplementation did not significantly affect the *α*- or *β*-diversity of the gut microbiota, suggesting that betaine supplementation had limited effects on overall microbial community structure at the compositional level. Instead, its effects may be mainly reflected in fine-scale regulation at specific taxonomic and potential functional levels. This was in agreement with several studies reporting that nutritional interventions often have limited effects on overall microbial diversity, likely due to functional redundancy within the gut microbiome (22).

In the low-dose betaine group, several genera associated with carbohydrate utilization, including *Prevotellaceae_NK3B31_group*, *unclassified_Bacteroidales*, and *Fibrobacter*, showed increased relative abundances. Previous studies have linked these taxa to dietary fiber and complex polysaccharide utilization. Therefore, their enrichment may suggest a potential shift in carbohydrate-utilization-related microbial composition. However, because microbial enzyme activity and fermentation products were not directly measured, this interpretation remains inferential (23–25).

Oscillibacter and Oscillospira were also enriched in the low-dose group. These taxa have been previously associated with fermentation-related microbial functions and SCFA production potential (26, 27). However, SCFAs were not measured in this study; therefore, their enrichment should be interpreted only as a possible microbiota-related feature associated with betaine supplementation.

The decreased abundance of Ruminococcus_torques_group in the low-dose group may also be noteworthy. This genus has been associated with mucin degradation and diarrhea-related intestinal conditions in previous studies (28, 29). Nevertheless, mucus layer integrity, intestinal barrier markers, and pathogen abundance were not measured in the present study. Therefore, whether the decrease in Ruminococcus_torques_group contributed to reduced diarrhea incidence remains to be validated.

PICRUSt2 and Tax4Fun2 predicted differences in several KEGG Level 3 pathways (20, 21), including citrate cycle (TCA cycle) (ko00020), folate biosynthesis (ko00790), lipoic acid metabolism (ko00785), and lipopolysaccharide biosynthesis (ko00540), and pentose and glucuronate interconversions (ko00040). These predicted pathways are broadly related to microbial carbohydrate metabolism, energy metabolism, and cofactor metabolism. However, because these functions were inferred from 16S rRNA gene sequencing data, they should not be interpreted as direct evidence of altered microbial metabolic activity. Therefore, these results should be considered predicted functional shifts and require validation by shotgun metagenomics, metatranscriptomics, metabolomics, or targeted SCFA measurement.

Exploratory correlation analysis showed that *norank_Selenomonadaceae*, *unclassified_Selenomonadaceae*, and *norank_UCG-010* were significantly positively associated with predicted ko00650, ko00020, and ko00040. These associations suggest that these genera may co-vary with predicted carbohydrate and energy metabolism-related pathways under betaine supplementation. Members of the family *Selenomonadaceae* are typical anaerobic fermenters with strong carbohydrate-fermenting capacity, capable of producing intermediate metabolites such as acetate and lactate that may participate in cross-feeding interactions. Therefore, their positive associations with predicted metabolic pathways may indicate possible links with carbohydrate fermentation and carbon-flow-related microbial functions, although this inference requires validation by metagenomic or metabolomic analyses. In addition, *Oscillibacter* and *Oscillospira* were also positively correlated with the ko00020. These taxa are commonly associated with fiber fermentation and short-chain fatty acid production potential. These observations suggest a possible association between betaine supplementation and fermentative microbial potential, but they do not demonstrate enhanced carbon utilization efficiency directly.

Interestingly, the phenotypic and microbiota-related responses were not completely dose-dependent. Both low-dose and high-dose betaine supplementation improved ADG, FCR, and diarrhea incidence, and the high-dose group showed an even lower diarrhea incidence than the low-dose group. However, most changes in bacterial genera and predicted KEGG pathways were more evident in the low-dose group. This inconsistency suggests that the observed colonic microbial changes cannot fully explain the overall improvement in growth performance and diarrhea incidence, particularly in the high-dose group. Therefore, the beneficial effects of betaine may also involve microbiota-independent or host-related mechanisms, such as methyl-donor metabolism, osmotic regulation, nutrient utilization, antioxidant responses, or intestinal physiological adaptation.

Several limitations should be acknowledged. First, microbiome analyses were conducted using five biological replicates per treatment, with one representative piglet selected from each pen. Although this sampling strategy avoided pseudo-replication at the pen level, the relatively small microbiome sample size may limit statistical power and the generalizability of the microbial findings. Second, predicted KEGG pathways were inferred from 16S rRNA gene sequencing data using PICRUSt2 and Tax4Fun2; therefore, these results represent predicted functional potential rather than directly measured microbial gene abundance, gene expression, enzyme activity, or metabolite production. Third, SCFAs, microbial metabolites, intestinal barrier markers, immune indicators, mucin layer integrity, and pathogen abundance were not directly measured. Finally, the limited sample size may affect the robustness of the random forest model and increase the risk of overfitting. Therefore, the microbiota-related findings should be interpreted as associative and hypothesis-generating and require validation in larger independent cohorts.

## 5 Conclusion

In conclusion, dietary betaine supplementation at 1250 and 2,500 mg/kg improved growth performance and reduced diarrhea incidence in weaned piglets, without significantly altering overall colonic microbial diversity. Several bacterial genera and predicted KEGG pathways differed among treatments, particularly in the low-dose betaine group, suggesting that betaine supplementation may be associated with changes in colonic microbial composition and predicted functional potential. However, because SCFAs, microbial metabolites, intestinal barrier function, and immune markers were not directly measured, these microbiota-related findings should be interpreted as associative and hypothesis-generating. Further studies integrating metagenomics, metabolomics, SCFA measurement, and host intestinal physiological indicators are needed to clarify the mechanisms underlying the beneficial effects of betaine in weaned piglets.

## Data Availability

The datasets presented in this study can be found in online repositories. The names of the repository/repositories and accession number(s) can be found in the article/Supplementary material.

## References

[1] Saladrigas-García M D'Angelo M Ko HL Nolis P Ramayo-Caldas Y Folch JM . Understanding host-microbiota interactions in the commercial piglet around weaning. Sci Rep. (2021) 11:23488. doi: 10.1038/s41598-021-02754-6 PMC8648723 34873196

[2] Upadhaya SD Kim IH. The impact of weaning stress on gut health and the mechanistic aspects of several feed additives contributing to improved gut health function in weanling piglets-a review. Animals. (2021) 11:11082418. doi: 10.3390/ani11082418, 34438875 PMC8388735

[3] Zheng L Duarte ME Sevarolli Loftus A Kim SW. Intestinal health of pigs upon weaning: challenges and nutritional intervention. Front Vet Sci. (2021) 8:628258. doi: 10.3389/fvets.2021.628258, 33644153 PMC7906973

[4] Wei X Tsai T Howe S Zhao J. Weaning induced gut dysfunction and nutritional interventions in nursery pigs: a partial review. Animals. (2021) 11:1279. doi: 10.3390/ani11051279, 33946901 PMC8146462

[5] Fan Z Lei L Wu X Xing R Du P Wang Z . Dietary fatty acids promote gut health in weaned piglets by regulating gut microbiota and immune function. Front Microbiol. (2025) 16:1558588. doi: 10.3389/fmicb.2025.1558588 PMC12014538 40270814

[6] Fang X Wang Z Chen Q Du Y Sun H Liu H . Protective effect of the branched short-chain fatty acid isobutyrate on intestinal damage in weaned piglets through intestinal microbiota remodeling. J Sci Food Agric. (2025) 105:1556–68. doi: 10.1002/jsfa.13930 39412364

[7] Eklund M Bauer E Wamatu J Mosenthin R. Potential nutritional and physiological functions of betaine in livestock. Nutr Res Rev. (2005) 18:31–48. doi: 10.1079/NRR200493, 19079893

[8] Xiong L Wang K Song M Azad MAK Zhu Q Kong X. Dietary betaine supplementation enhances colonic barrier function through the Nrf2/Keap1 and TLR4-NF-κB/MAPK Signaling pathways and alters colonic microbiota in Bama Mini-pigs. Antioxidants (Basel). (2023) 12:1926. doi: 10.3390/antiox12111926, 38001779 PMC10669150

[9] Fu R Liang C Chen D Tian G Zheng P He J . Effects of low-energy diet supplemented with betaine on growth performance, nutrient digestibility, and serum metabolomic profiles in growing pigs. J Anim Sci. (2023) 101:skad080. doi: 10.1093/jas/skad080, 36930062 PMC10066726

[10] Wang H Li S Xu S Feng J. Betaine improves growth performance by increasing digestive enzymes activities, and enhancing intestinal structure of weaned piglets. Anim Feed Sci Technol. (2020) 267:114545. doi: 10.1016/j.anifeedsci.2020.114545

[11] Pardo Z Mateos I Saro C Campos R Argüello H Lachica M . The effect of supplementation with betaine and zinc on in vitro large intestinal fermentation in Iberian pigs under heat stress. Animals. (2023) 13. doi: 10.3390/ani13061102, 36978642 PMC10044697

[12] National Research Council. Nutrient Requirements of Swine: Eleventh Revised Edition. Washington, DC: The National Academies Press (2012).

[13] Pedersen KS Toft N. Intra- and inter-observer agreement when using a descriptive classification scale for clinical assessment of faecal consistency in growing pigs. Prev Vet Med. (2011) 98:288–91. doi: 10.1016/j.prevetmed.2010.11.016, 21185096

[14] Moeser AJ Klok CV Ryan KA Wooten JG Little D Cook VL . Stress signaling pathways activated by weaning mediate intestinal dysfunction in the pig. Am J Physiol Gastrointest Liver Physiol. (2007) 292:G173–81. doi: 10.1152/ajpgi.00197.2006 16901995

[15] Martin M. CUTADAPT removes adapter sequences from high-throughput sequencing reads. EMBnetjournal. (2011) 17:10. doi: 10.14806/ej.17.1.200

[16] Zhang J Kobert K Flouri T Stamatakis A. PEAR: a fast and accurate Illumina paired-end reAd mergeR. Bioinformatics. (2014) 30:614–20. doi: 10.1093/bioinformatics/btt593, 24142950 PMC3933873

[17] Schmieder R Edwards R. Quality control and preprocessing of metagenomic datasets. Bioinformatics. (2011) 27:863–4. doi: 10.1093/bioinformatics/btr026, 21278185 PMC3051327

[18] Edgar RC. UPARSE: highly accurate OTU sequences from microbial amplicon reads. Nat Methods. (2013) 10:996–8. doi: 10.1038/nmeth.2604, 23955772

[19] Wang Q Garrity GM Tiedje JM Cole JR. Naive Bayesian classifier for rapid assignment of rRNA sequences into the new bacterial taxonomy. Appl Environ Microbiol. (2007) 73:5261–7. doi: 10.1128/aem.00062-07, 17586664 PMC1950982

[20] Douglas GM Maffei VJ Zaneveld JR Yurgel SN Brown JR Taylor CM . PICRUSt2 for prediction of metagenome functions. Nat Biotechnol. (2020) 38:685–8. doi: 10.1038/s41587-020-0548-6, 32483366 PMC7365738

[21] Wemheuer F Taylor JA Daniel R Johnston E Meinicke P Thomas T . Tax4Fun2: prediction of habitat-specific functional profiles and functional redundancy based on 16S rRNA gene sequences. Environ Microbiome. (2020) 15:11. doi: 10.1186/s40793-020-00358-7, 33902725 PMC8067651

[22] Moya A Ferrer M. Functional redundancy-induced stability of gut microbiota subjected to disturbance. Trends Microbiol. (2016) 24:402–13. doi: 10.1016/j.tim.2016.02.002, 26996765

[23] Amat S Lantz H Munyaka PM Willing BP. Prevotella in pigs: the positive and negative associations with production and health. Microorganisms. (2020) 8:1584. doi: 10.3390/microorganisms8101584, 33066697 PMC7602465

[24] Grondin JM Tamura K Déjean G Abbott DW Brumer H. Polysaccharide Utilization Loci: Fueling Microbial Communities. J Bacteriol. (2017) 199:e00860-16. doi: 10.1128/jb.00860-16, 28138099 PMC5512228

[25] Suen G Weimer PJ Stevenson DM Aylward FO Boyum J Deneke J . The complete genome sequence of *Fibrobacter succinogenes* S85 reveals a cellulolytic and metabolic specialist. PLoS One. (2011) 6:e18814. doi: 10.1371/journal.pone.0018814, 21526192 PMC3079729

[26] Louis P Flint HJ. Formation of propionate and butyrate by the human colonic microbiota. Environ Microbiol. (2017) 19:29–41. doi: 10.1111/1462-2920.13589, 27928878

[27] Yang J Li Y Wen Z Liu W Meng L Huang H. Oscillospira - a candidate for the next-generation probiotics. Gut Microbes. (2021a) 13:1987783. doi: 10.1080/19490976.2021.1987783, 34693878 PMC8547878

[28] Desai MS Seekatz AM Koropatkin NM Kamada N Hickey CA Wolter M . A dietary Fiber-deprived gut microbiota degrades the colonic mucus barrier and enhances pathogen susceptibility. Cell. (2016) 167:1339–1353.e21. doi: 10.1016/j.cell.2016.10.043, 27863247 PMC5131798

[29] Huang T Dong J Zhang W Hu Z Tan X Li H . Comparison of fecal microbiota and metabolites in diarrheal piglets pre-and post-weaning. Front Vet Sci. (2025) 12:1613054. doi: 10.3389/fvets.2025.1613054 PMC12226869 40621496

