# Supplementary material for: Effects of betaine on growth performance and colonic microbiota in weaned piglets
Source: Front Vet Sci. 2026 Oct 28;13:1937273. doi: 10.3389/fvets.2026.1937273 (PMC13751383; doi:10.3389/fvets.2026.1937273)
Supplement: Supplementary file 1 [file Data_sheet_1.docx]

Supplementary Material

# 1 Supplementary Figures and Tables

## 1.1 Supplementary Figures

**Supplementary Figure 1. Effects of dietary betaine supplementation on alpha and beta diversity of colonic microbiota in weaned piglets.**

(A) Box plots showing alpha diversity indices of colonic microbiota across the three treatment groups, including Observed species (S.obs), Chao1 richness estimator, Shannon index, and Simpson index.
(B) Principal coordinate analysis (PCoA) plot based on Bray-Curtis distance, with each point representing an individual pig (n = 5 per group). CON, basal diet; Low_Dose, basal diet supplemented with 1250 mg/kg betaine; High_Dose, basal diet supplemented with 2500 mg/kg betaine.

**Supplementary Figure 2. Relative abundances of additional microbial genera identified by random forest analysis across experimental groups.**

Data are presented as mean ± standard error (SE). Asterisks indicate significant differences (* *P* < 0.05, ** *P* < 0.01), and ns indicates not significant (*P* > 0.05).

## 1.2 **Su**pplementary Tables

## **Supplementary** Table 1. Ingredient composition and nutrient content of the base diets

| Ingredient | Content (%) | Nutrient | Content |
| --- | --- | --- | --- |
| Corn | 60.00 | Digestible energy (DE, MJ/kg) | 14.24 |
| Soybean meal | 20.00 | Crude protein (CP, %) | 18.82 |
| Fish meal | 4.50 | Ether extract (EE, %) | 6.23 |
| Wheat bran | 3.00 | Calcium (Ca, %) | 0.77 |
| Soybean oil | 1.90 | Total phosphorus (P, %) | 0.61 |
| Whey powder | 5.00 | Lysine (Lys, %) | 1.28 |
| CaHPO₄ | 1.30 | Methionine (Met, %) | 0.41 |
| Yeast | 2.00 | Threonine (Thr, %) | 0.83 |
| NaCl | 0.30 | Tryptophan (Trp, %) | 0.25 |
| Premix¹ | 2.00 |  |  |
| Total | 100.00 |  |  |

¹ The premix provided per kilogram of diet: vitamin A 8,500 IU; vitamin D₃ 3,500 IU; vitamin E 20 IU; vitamin K₃ 2.0 mg; vitamin B₁ 1.5 mg; vitamin B₂ 4.0 mg; vitamin B₆ 3.5 mg; vitamin B₁₂ 0.2 mg; nicotinic acid 30.0 mg; folic acid 0.85 mg; pantothenic acid 15.0 mg; biotin 0.1 mg; Fe 100 mg; Cu 150 mg; Mn 20 mg; Zn 100 mg; I 0.3 mg; Se 0.3 mg.
